# Cognitive impairment and dependency in activities of daily living: a cross-lagged analysis

**DOI:** 10.3389/fnagi.2025.1663237

**Published:** 2025-12-08

**Authors:** Xuewei Fu, Shuming Ji, Jiafeng Li, Ting Chen, Xuhuan Liu, Weihong Kuang

**Affiliations:** 1 Mental Health Center, National Center for Mental Disorders, West China Hospital, Sichuan University, Chengdu, China; 2 Department of Clinical Research Management, West China Hospital of Sichuan University, Chengdu, China

**Keywords:** cognitive impairment, activities of daily living, cognition, cross-lagged analysis, longitudinal study

## Abstract

**Background:**

This study investigates the bidirectional relationship between cognitive function and Activities of Daily Living (ADL) at varying levels of cognitive impairment. Specifically, it explores how cognitive function affects Basic Activities of Daily Living (BADL) and Instrumental Activities of Daily Living (IADL) over time, and how changes in ADL impact cognitive function.

**Method:**

A retrospective analysis of clinical data was conducted using cross-lagged panel models to examine the relationships between cognitive function and ADL. Cognitive function was measured using the Montreal Cognitive Assessment, while ADL were assessed via standardized measures of both BADL and IADL at two time points. Participants were grouped according to the severity of their initial cognitive impairment, allowing for comparative analysis across different levels of impairment.

**Results:**

Cross-lagged analyses revealed that lower baseline cognitive function was significantly associated with increased ADL dependency at follow-up (*β* = −0.302, *p* < 0.001), with a stronger association for IADL (*β* = −0.318, *p* < 0.001) than for BADL (β = −0.274, *p* < 0.001). Subgroup analyses indicated that these associations were most pronounced in individuals with moderate cognitive impairment.

**Conclusion:**

This study highlights the significant association between cognitive function and ADL, with lower baseline cognitive function predicting increased dependency, particularly in IADL. The findings emphasize the importance of early cognitive health interventions to prevent further decline in daily functioning, especially in individuals with moderate cognitive impairment.

## 1 Introduction

Activities of Daily Living (ADL) refer to essential tasks necessary for everyday functioning and are critical for maintaining independence and quality of life (Giebel et al., 2014; Gobbens, 2018). Cognitive impairment, a prevalent condition among older adults, affects millions worldwide, with approximately 15–20% of this population experiencing some form of cognitive decline (Alzheimer's Association, 2023). As cognitive abilities deteriorate, individuals often face challenges in performing ADL (Romero-Ayuso et al., 2021), which can adversely affect their well-being and autonomy. This decline also imposes significant burdens on caregivers, potentially diminishing their quality of life and increasing caregiver strain (Sung et al., 2022). Therefore, understanding the impact of cognitive impairment on ADL is vital for developing effective caregiving and intervention strategies aimed at improving overall quality of life.

Given the complexity of both ADL and cognitive function, their relationship is inherently multifaceted. ADL comprise two components: Basic Activities of Daily Living (BADL), such as bathing, dressing, and eating, which are often guided by habits and routines; and Instrumental Activities of Daily Living (IADL), encompassing more complex tasks like using public transportation, managing finances, or shopping (Katz, 1963; Lawton and Brody, 1969). Research studies suggest that while both components are associated with cognitive function (Millán-Calenti et al., 2012; Zhang and Yang, 2024), IADL are more vulnerable to early cognitive impairment compared to BADL (Clemmensen et al., 2020; Jekel et al., 2015). However, most studies have primarily focused on individuals with Mild Cognitive Impairment (MCI) (Jekel et al., 2015; Lindbergh et al., 2016; Montoro-Membila et al., 2022), with limited attention to how cognitive decline affects ADL in those with moderate to severe impairment. This gap constrains our understanding of how BADL and IADL are impacted across different stages, limiting the development of effective interventions. This study addresses these gaps by examining the relationship between cognitive function and ADL across varying levels of cognitive impairment.

Moreover, the direction of the relationship between cognitive function and ADL remains unclear. While cognitive decline can impair the ability to perform daily activities, reductions in ADL engagement may also contribute to further cognitive deterioration through decreased stimulation and functional practice (Weaver and Jaeggi, 2021). To address this complexity, a cross-lagged panel model was employed, which enables the simultaneous estimation of reciprocal associations between two variables measured at multiple time points, thereby providing a clearer picture of temporal precedence. Using a cross-lagged design, it explores the bidirectional dynamics between cognitive function and ADL over time, focusing on the temporal association between cognitive function and ADL.

By distinguishing between different components of ADL and examining individuals across varying levels of cognitive impairment through a cross-lagged panel, this study explores how cognition and daily functioning is associated with each other over time at different levels of cognitive severity and across distinct ADL domains, allowing for a nuanced understanding of their bidirectional interplay.

## 2 Materials and methods

### 2.1 Data source and participants

From June 2016 to April 2024, data were obtained from the Assessment and Treatment Center for Clinical Psychology in West China Hospital. The participants included patients referred by physicians for assessments of cognitive function and ADL from both outpatient and inpatient departments. A total of 4,418 cases were initially extracted, encompassing both MoCA and ADL measurements. Among these, we identified 464 cases with repeated assessments. For patients who underwent more than two evaluations, only the first two time assessments (T1 and T2) were selected, resulting in a dataset of 440 cases, corresponding to 220 patients. The time interval between assessments for each patient was at least 6 months to minimize learning effects from retesting the MoCA, and within 36 months to capture changes within an expected timeframe. Ultimately, 169 participants were included in the study. The participants were drawn from a clinical population referred for cognitive and functional assessments, and therefore have presented with a variety of neurological or psychiatric conditions; detailed diagnostic information was not consistently available. This study was approved by the Ethics Committee of West China Hospital of Sichuan University.

### 2.2 Measurement

#### 2.2.1 Cognitive function

Cognitive function was assessed using the Montreal Cognitive Assessment (MoCA), a validated tool designed to evaluate multiple cognitive domains, including short-term memory, visuospatial abilities, executive function, attention, concentration, working memory, language, and orientation (Nasreddine et al., 2005). The MoCA includes 30 items, with total scores ranging from 0 to 30; lower scores indicate more severe cognitive impairment. Based on T1 MoCA scores and classification criteria from the MoCA official guidelines, participants were grouped into three categories: normal/mild impairment (scores > 17, *n* = 39), moderate impairment (scores 10–17, *n* = 97), and severe impairment (scores < 0, *n* = 33). All assessments were conducted in quiet environments by trained healthcare professionals to ensure consistency in testing and scoring.

#### 2.2.2 ADL dependency

Dependency in ADL was assessed through interviews with caretakers (e.g., partners, siblings, or children). The assessment includes 14 items categorized into BADL and IADL (Katz, 1963; Lawton and Brody, 1969). BADL consists of six essential activities: ambulating, feeding, dressing, toileting, grooming, and bathing. IADL includes more complex tasks, including using transportation, preparing meals, housekeeping, taking medications, doing laundry, shopping, using the telephone, and managing finances. Each item is scored from 1 to 4, yielding a total score range of 14 to 64, with higher scores indicating greater dependency on assistance for daily activities.

### 2.3 Covariates

Age and sex at T1 were included as covariates in the cross-lagged panel analyses due to their potential impact on cognitive function and ADL. The time interval between T1 and T2, calculated in months, was also controlled to account for its influence on observed changes.

### 2.4 Statistical analysis

Bivariate correlation analyses were performed to examine the relationships between cognitive function (MoCA) and ADL domains (total ADL, BADL, and IADL) both across and within time points. Correlations were calculated for the total sample as well as for subgroups stratified by cognitive impairment severity to explore potential differential patterns. This approach provided a preliminary overview of the associations before conducting cross-lagged panel analyses. Descriptive statistics and correlation analyses were conducted using IBM SPSS Statistics 29.0.

Cross-lagged panel models were used to analyze the bidirectional relationships between cognitive function and ADL over time. These analyses were initially performed for the total sample, focusing separately on total ADL, BADL, and IADL. Further subgroup analyses were conducted based on cognitive impairment severity (normal/mild, moderate, and severe), yielding nine models in total. These models assessed how cognitive function at T1 was associated with ADL dependency at T2, and vice versa. Age, sex, and the time interval between assessments were incorporated as covariates to control for confounding effects. Cross-lagged models were estimated using Mplus 8.0, and model fit was evaluated using several indices: the Comparative Fit Index (CFI) and Tucker-Lewis Index (TLI), where values >0.90 indicated good fit; the Root Mean Square Error of Approximation (RMSEA), with values <0.08 considered acceptable; and the Standardized Root Mean Square Residual (SRMR), where values <0.08 suggested good fit. All analyses were conducted using two-tailed tests, and *p*-values < 0.05 were considered statistically significant.

## 3 Results

### 3.1 Description of the participants

Among the 169 participants who completed both measurements, 46.7% were male (79 individuals) and 53.3% were female (90 individuals). Ages ranged from 47 to 88 years, with a mean of 71.54 years (SD = 8.57). A comparison between the two time points revealed a significant decline in cognitive function, evidenced by a decrease in MoCA scores from T1 to T2 (mean difference = −1.80, *t*(168) = −6.06, *p* < 0.001, 95% CI [−2.39, −1.21], Cohen’s *d* = 0.47). Concurrently, ADL scores, including BADL and IADL, increased significantly, reflecting greater dependency in daily activities over time: total ADL (mean difference = 4.17, *t*(168) = 6.73, *p* < 0.001, 95% CI [2.95, 5.40], *d* = 0.52), BADL (mean difference = 1.12, *t*(168) = 5.07, *p* < 0.001, 95% CI [0.68, 1.56], *d* = 0.39), and IADL (mean difference = 3.05, *t*(168) = 6.82, *p* < 0.001, 95% CI [2.17, 3.94], *d* = 0.52). These findings suggest that participants experienced declining cognitive abilities and increasing dependence in daily life over the study period. Detailed data are presented in Table 1.

**Table 1:** Characteristics of all participants at baseline (T1) and follow-up (T2).

Variables	T1		T2		T2-T1(month)			
	Number	Percentage	Number	Percentage	Mean	SD	Min	Max
**Gender**					17.54	7.54	6.03	35.83
Male	79	46.7%	79	46.7%				
Female	90	53.3%	90	53.3%				

	**Mean**	**SD**	**Mean**	**SD**	**Mean Difference (95% CI)**	***t*(df = 168)**	***p***	**Cohen’s *d***
**Age**	71.54	8.574	72.71	8.510	1.172 [1.04, 1.31]	16.927**	<0.01	1.30
**MoCA**	13.94	5.173	12.14	6.163	−1.799 [−2.39, −1.21]	−6.058**	<0.01	0.47
**ADL**	21.38	6.976	25.55	9.393	4.172 [2.95, 5.40]	6.730**	<0.01	0.52
**BADL**	7.20	2.192	8.32	3.221	1.118 [0.68, 1.55]	5.070**	<0.01	0.39
**IADL**	14.18	5.318	17.23	6.636	3.053 [2.17, 3.94]	6.815**	<0.01	0.52

SD, Standard deviation; Mean difference indicates the change in scores from T1 to T2; higher MoCA scores reflect better cognitive performance, while higher ADL, BADL, and IADL scores indicate greater dependence in daily activities; *p < 0.05, ** *p* < 0.01, and *** *p* < 0.001.

### 3.2 Correlations between MoCA and ADLs

The correlation analysis reveals significant relationships between cognitive function and ADLs. In the total sample, MoCA scores were negatively correlated with ADL scores concurrently at both time points (T1: *r* = −0.478, *p* < 0.01; T2: *r* = −0.589, *p* < 0.01). Longitudinally, MoCA at T1 (MoCA1) was negatively correlated with ADL at T2 (ADL2) (*r* = −0.487, *p* < 0.01), while ADL at T1 (ADL1) was negatively correlated with MoCA at T2 (MoCA2) (*r* = −0.294, *p* < 0.01). Similar trends were observed for ADL subdomains: MoCA1 correlated negatively with BADL at T2 (BADL2) (*r* = −0.389, *p* < 0.01) and IADL at T2 (IADL2) (*r* = −0.500, *p* < 0.01). Regarding the reverse direction, BADL at T1 (BADL1) was not significantly correlated with MoCA2 (*r* = −0.070, *p* = 0.369), whereas IADL at T1 (IADL1) showed a significant negative association with MoCA2 (*r* = −0.357, *p* < 0.01). Detailed correlations are summarized in Table 2.

**Table 2:** Correlations between cognitive function and ADLs at baseline (T1) and follow-up (T2).

Variables	**MoCA1**	**ADL1**	**BADL1**	**IADL1**	**MoCA2**	**ADL2**	**BADL2**	**IADL2**
**MoCA1**	-							
**ADL1**	−0.478**	-						
**BADL1**	−0.302**	0.824**	-					
**IADL1**	−0.502**	0.972**	0.668**	-				
**MoCA2**	0.782**	−0.294**	−0.070	−0.357**	-			
**ADL2**	−0.487*	0.549*	0.389**	0.560*	−0.589**	-		
**BADL2**	−0.389**	0.545**	0.493**	0.512**	−0.460*	0.902**	-	
**IADL2**	−0.500**	0.513**	0.312**	0.544**	−0.611*	0.978**	0.791**	-

Correlations are Pearson correlation coefficients; MoCA1, MoCA at T1; ADL1, ADL at T1; BADL1, BADL at T1; IADL1, IADL at T1; MoCA2, MoCA at T2; ADL2, ADL at T2; BADL2, BADL at T2; IADL2, IADL at T2; * *p* < 0.05, ** *p* < 0.01, and *** *p* < 0.001.

When examined by cognitive subgroup, these patterns were generally consistent but varied across cognitive impairment severity. The negative associations between MoCA1 and ADL2 remained significant in both the normal/mild (*r* = −0.346, *p* < 0.05) and moderate groups (*r* = −0.350, *p* < 0.05), but were non-significant in the severe group (*r* = −0.311, *p* = 0.078). Similar trends were observed for IADL2, which was negatively correlated with MoCA1 in the normal/mild (*r* = −0.372, *p* < 0.05), moderate (*r* = −0.320, *p* < 0.01), but was not in severe groups (*r* = −0.327, *p* = 0.063), whereas associations between MoCA1 and BADL2 were weaker and less consistent, reaching significance only in the moderate group (*r* = −0.362, *p* < 0.01). In the reverse direction, baseline ADLs showed no significant associations with MoCA2 in any subgroup, except for BADL1 in the severe group, which was positively correlated with MoCA2 (*r* = 0.468, *p* < 0.01). Subgroup correlations details are provided in the Supplementary material (Table 1S).

### 3.3 Cross-lagged panel analyses

The cross-lagged panel model demonstrated good fit for the total sample and subgroups. For the total sample, the chi-square value was non-significant (*χ*^2^ = 11.11, *p* = 0.085), with CFI and TLI values >0.95, and an RMSEA of 0.071. Subgroup models also showed acceptable fit. Detailed indices are available in the Supplementary material (Table 2S).

**Figure 1:**
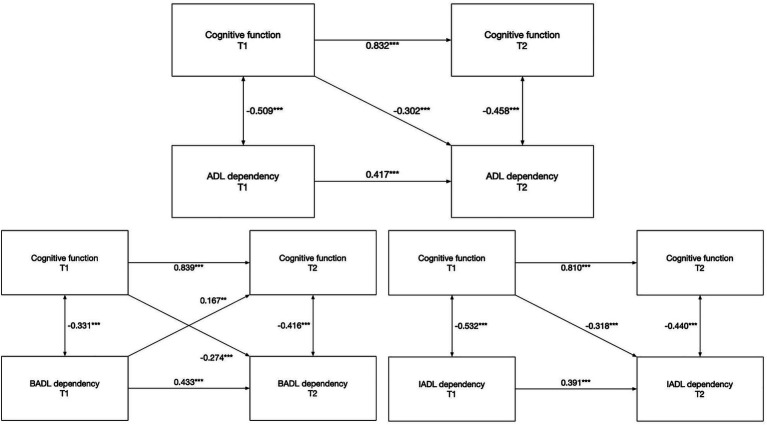
Cross-lagged models of cognitive function and ADLs. The covariates are not shown here to simplify the representation of the model; * *p* < 0.05, **p < 0.01, and ***p < 0.001.

The cross-lagged analysis (Figure 1) highlighted significant relationships between cognitive function and ADL. In the total sample, MoCA1 had a significant negative association with ADL2 (*β* = −0.302, *p* < 0.01), indicating that poorer cognitive function at baseline was associated with greater dependency at follow-up. In contrast, the path from ADL1 to MoCA2 was non-significant (*β* = 0.094, *p* = 0.073).

When analyzing BADL and IADL separately, MoCA1 was negatively associated with both IADL2 (*β* = −0.318, *p* < 0.001) and BADL2 (*β* = −0.274, *p* < 0.001), which are consistent with the overall ADL result. Additionally, BADL1 demonstrated a significant positive path to MoCA2 (*β* = 0.167, *p* < 0.001), suggesting that greater dependency in basic activities at the initial assessment is linked to improved cognitive function at follow-up.

Subgroup analyses revealed nuanced patterns. In the normal/mild group, no significant cross-lagged paths were found. In the moderate impairment group, MoCA1 was negatively associated with ADL2 (*β* = −0.244, *p* < 0.001), BADL2 (*β* = −0.316, *p* < 0.001), and IADL2 (*β* = −0.202, *p* < 0.05). In the severe group, MoCA1 was significantly associated with ADL2 and BADL2 but not IADL2, while BADL1 had a positive association with MoCA2 (*β* = 0.398, *p* < 0.001). The results for all cross-lagged paths are presented in Table 3.

**Table 3:** Cross-lagged paths of MoCA and ADLs across different cognitive impairment groups.

Model path	**Total sample**		**Normal/mild group**		**Moderate group**		**Severe group**	
	*β*	***p***	*β*	***p***	*β*	***p***	*β*	***p***
**MoCA1 → ADL2**	−0.302***	<0.001	−0.258	0.060	−0.244**	0.009	−0.242*	0.028
**ADL1 → MoCA2**	0.094	0.073	0.137	0.201	0.042	0.673	0.224	0.059
**MoCA1 → IADL2**	−0.318***	<0.001	−0.260	0.057	−0.202*	0.037	−0.234	0.056
**IADL1 → MoCA2**	0.044	0.405	0.122	0.266	−0.004	0.965	0.094	0.447
**MoCA1 → BADL2**	−0.274***	<0.001	−0.191	0.178	−0.316***	<0.001	−0.246*	0.029
**BADL1 → MoCA2**	0.167***	0.001	0.145	0.166	0.164	0.078	0.398***	<0.001

*β*, standardized coefficient; * *p* < 0.05, ** *p* < 0.01, and *** *p* < 0.001.

## 4 Discussion

This study provides robust evidence for the intricate relationship between cognitive function and ADL. The overall cross-lagged analysis revealed that lower baseline cognitive function was significantly associated with greater ADL dependency at follow-up, whereas the opposite path—from baseline ADL to later cognition—was not significant, which suggests that cognitive decline may precede and contribute to subsequent functional deterioration rather than result from it, which is consistent with prior research showing that cognitive impairment often precedes functional limitations (Martin et al., 2023; Sun et al., 2022; Zahodne et al., 2013).

Further analysis of ADL components revealed significant negative associations between baseline cognitive function (MoCA) and follow-up IADL and BADL. These results suggest that cognitive decline is broadly associated with dependency across various daily tasks, with more complex IADLs being particularly vulnerable to early cognitive deterioration, consistent with previous studies (Nygård, 2003; Reppermund et al., 2013).

Subgroup analyses revealed distinct patterns across cognitive impairment levels. In the normal/mild impairment group, no significant cross-lagged paths were found, reflecting that at early stages of cognitive decline, daily functioning may not be substantially associated with prior cognitive changes, which aligns with studies reporting preserved ADL function in mild cognitive impairment (Perneczky et al., 2006). In the moderate impairment group, baseline cognitive function was negatively associated with follow-up ADL, IADL, and BADL, suggesting that declines in cognition tend to precede greater dependency in both complex and basic tasks. Among individuals with severe impairment, baseline cognitive function (MoCA1) was negatively associated with ADL2 and BADL2, while the path to IADL2 was not significant. These findings underscore the progressive association of cognitive decline with dependency, particularly in moderate and severe impairment groups.

Interestingly, the severe impairment group also exhibited a significant positive path from baseline BADL dependency to follow-up cognitive function, consistent with findings from the overall sample. This suggests that rehabilitative care and social support may stabilize or modestly enhance cognitive function (Butsing et al., 2024; Ham et al., 2021). However, the less favorable fit indices for the severe impairment group suggest that the model may not fully capture the complexity of these dynamics, highlighting the need for further exploration of contextual factors influencing these relationships (Zeng et al., 2024).

Several limitations must be noted in this study. First, the sample was drawn from a single hospital, which resulted in relatively small subgroup sizes within each cognitive impairment category. Consequently, the statistical power in some subgroups may have been insufficient to detect significant cross-lagged paths, particularly in the normal/mild and moderate impairment groups. It is also important to acknowledge limitations inherent to cross-lagged panel model, such as potential inflated error rates when multiple models are tested (Lucas, 2023), given that nine different models were examined in this study. Additionally, ADL assessments relied on caregiver reports, which could be influenced by varying perceptions of the functional abilities. Furthermore, the complexity of the relationships, especially in the severe impairment group, suggests that the current model may not fully capture all the nuances involved. Future research could incorporate qualitative methods to explore these contextual influences. Nevertheless, this study lays important groundwork for understanding the progression of cognitive function and ADL dependency, offering a basis for further research and targeted interventions.

## 5 Conclusion

In conclusion, this study underscores that cognitive decline tends to precede increases in ADL dependency, particularly highlighting that IADLs are more vulnerable to early cognitive changes than BADLs. The cross-lagged analysis revealed distinct patterns across various levels of cognitive impairment, suggesting that interventions should be tailored to the cognitive status of individuals. Future studies could employ larger, multi-center samples and integrate objective functional assessments to complement caregiver reports. Moreover, longitudinal studies that include intervention could further clarify the mechanisms linking cognitive decline and functional dependency.

## Data Availability

The raw data that support the findings of this study are held by West China Hospital of Sichuan University. Access may be granted upon reasonable request, subject to approval in accordance with the hospital’s data management policies.
