# Supplementary material for: Cognitive impairment and dependency in activities of daily living: a cross-lagged analysis
Source: Front Aging Neurosci. 2025 Dec 8;17:1663237. doi: 10.3389/fnagi.2025.1663237 (PMC12719466; doi:10.3389/fnagi.2025.1663237)
Supplement: Supplementary file 1 [file Table_1.DOCX]

# Supplementary Materials

## Table 1S. Correlations of MoCA and ADLs in three cognitive impairment groups

|  | **MoCA1** | **ADL1** | **BADL1** | **IADL1** | **MoCA2** | **ADL2** | **BADL2** | **IADL2** |
| --- | --- | --- | --- | --- | --- | --- | --- | --- |
| **Normal/Mild Group** |  |  |  |  |  |  |  |  |
| MoCA1 | - |  |  |  |  |  |  |  |
| ADL1 | -.190 | - |  |  |  |  |  |  |
| BADL1 | .022 | .768** | - |  |  |  |  |  |
| IADL1 | -.243 | .976** | .610** | - |  |  |  |  |
| MoCA2 | .709** | -.003 | .145 | -.053 | - |  |  |  |
| ADL2 | -.346* | .361* | .136 | .401* | -.408** | - |  |  |
| BADL2 | -.222 | .284 | .244 | .268 | -.371* | .874** | - |  |
| IADL2 | -.372* | .367* | .081 | .427** | -.393* | .979** | .757** | - |
| **Moderate Group** |  |  |  |  |  |  |  |  |
| MoCA1 | - |  |  |  |  |  |  |  |
| ADL1 | -.406** | - |  |  |  |  |  |  |
| BADL1 | -.248* | .760** | - |  |  |  |  |  |
| IADL1 | -.416** | .980** | .617** | - |  |  |  |  |
| MoCA2 | .405** | -.132 | .062 | -.179 | - |  |  |  |
| ADL2 | -.350* | .414* | .278** | .417** | -.575** | - |  |  |
| BADL2 | -.362** | .370** | .339** | .346** | -.480** | .901** | - |  |
| IADL2 | -.320** | .407** | .231* | .423** | -.580* | .979** | .794** | - |
| **Severe Group** |  |  |  |  |  |  |  |  |
| MoCA1 | - |  |  |  |  |  |  |  |
| ADL1 | -.050 | - |  |  |  |  |  |  |
| BADL1 | -.061 | .875** | - |  |  |  |  |  |
| IADL1 | -.115 | .952** | .685** | - |  |  |  |  |
| MoCA2 | .630** | .207 | .468** | .015 | - |  |  |  |
| ADL2 | -.311 | .619* | .466** | .637** | -.318 | - |  |  |
| BADL2 | -.249 | .689** | .594** | .662** | -.169 | .924** | - |  |
| IADL2 | -.327 | .518** | .339 | .565** | -.391* | .966** | .792** | - |

Notes: Correlations are Pearson correlation coefficients; * p < 0.05, **p < 0.01, and ***p < 0.001.

## Table 2S. Fit indices about cross-lagged panel models cognitive function and ADLs

| **Group** | **Model** | **χ2** | **df** | **p** | **CFI** | **TLI** | **RMSEA(90%CI)** | **SRMR** |
| --- | --- | --- | --- | --- | --- | --- | --- | --- |
| **Total Sample** | MoCA and ADL | 11.114 | 6 | 0.085 | 0.986 | 0.957 | 0.071  (0.000, 0.135) | 0.048 |
|  | MoCA and IADL | 10.551 | 6 | 0.103 | 0.987 | 0.961 | 0.067  (0.000, 0.132) | 0.045 |
|  | MoCA and BADL | 11.862 | 6 | 0.065 | 0.981 | 0.944 | 0.076  (0.000, 0.140) | 0.052 |
| **Normal/Mild Group** | MoCA and ADL | 3.127 | 6 | 0.793 | 1.000 | 1.226 | 0.000  (0.000, 0.135) | 0.045 |
|  | MoCA and IADL | 2.847 | 6 | 0.828 | 1.000 | 1.241 | 0.000  (0.000, 0.124) | 0.044 |
|  | MoCA and BADL | 3.791 | 6 | 0.705 | 1.000 | 1.190 | 0.000  (0.000, 0.157) | 0.047 |
| **Moderate Group** | MoCA and ADL | 12.218 | 6 | 0.057 | 0.937 | 0.812 | 0.103  (0.000, 0.187) | 0.053 |
|  | MoCA and IADL | 12.289 | 6 | 0.056 | 0.936 | 0.807 | 0.104  (0.000, 0.187) | 0.051 |
|  | MoCA and BADL | 10.471 | 6 | 0.106 | 0.941 | 0.824 | 0.088  (0.000, 0.174) | 0.059 |
| **Severe Group** | MoCA and ADL | 13.281 | 6 | 0.039 | 0.886 | 0.657 | 0.192  (0.041, 0.333) | 0.075 |
|  | MoCA and IADL | 10.476 | 6 | 0.106 | 0.910 | 0.729 | 0.150  (0.000, 0.298) | 0.071 |
|  | MoCA and BADL | 14.797 | 6 | 0.022 | 0.879 | 0.636 | 0.211  (0.075, 0.349) | 0.091 |

Notes: χ² = chi-square; df = degrees of freedom; CFI = Comparative Fit Index; TLI = Tucker–Lewis Index; RMSEA = Root Mean Square Error of Approximation; 90% CI = 90% Confidence Interval; SRMR = Standardized Root Mean Square Residual.
